# Environmental factors associated with the malaria vectors *Anopheles gambiae *and *Anopheles funestus *in Kenya

**DOI:** 10.1186/1475-2875-8-268

**Published:** 2009-11-26

**Authors:** Louise A Kelly-Hope, Janet Hemingway, F Ellis McKenzie

**Affiliations:** 1 Vector Group, Liverpool School of Tropical Medicine, Liverpool, UK; 2 Division of Epidemiology and Population Studies, Fogarty International Center, National Institutes of Health, Bethesda, MD, USA

## Abstract

**Background:**

The *Anopheles gambiae *and *Anopheles funestus *mosquito species complexes are the primary vectors of *Plasmodium falciparum *malaria in sub-Saharan Africa. To better understand the environmental factors influencing these species, the abundance, distribution and transmission data from a south-eastern Kenyan study were retrospectively analysed, and the climate, vegetation and elevation data in key locations compared.

**Methods:**

Thirty villages in Malindi, Kilifi and Kwale Districts with data on *An. gambiae sensu strict*, *Anopheles arabiensis* and *An. funestus* entomological inoculation rates (EIRs), were used as focal points for spatial and environmental analyses. Transmission patterns were examined for spatial autocorrelation using the Moran's *I *statistic, and for the clustering of high or low EIR values using the Getis-Ord Gi* statistic. Environmental data were derived from remote-sensed satellite sources of precipitation, temperature, specific humidity, Normalized Difference Vegetation Index (NDVI), and elevation. The relationship between transmission and environmental measures was examined using bivariate correlations, and by comparing environmental means between locations of high and low clustering using the Mann-Whitney *U *test.

**Results:**

Spatial analyses indicated positive autocorrelation of *An. arabiensis *and *An. funestus *transmission, but not of *An. gambiae s.s*., which was found to be widespread across the study region. The spatial clustering of high EIR values for *An. arabiensis *was confined to the lowland areas of Malindi, and for *An. funestus *to the southern districts of Kilifi and Kwale. Overall, *An. gambiae s.s*. and *An. arabiensis *had similar spatial and environmental trends, with higher transmission associated with higher precipitation, but lower temperature, humidity and NDVI measures than those locations with lower transmission by these species and/or in locations where transmission by *An. funestus *was high. Statistical comparisons indicated that precipitation and temperatures were significantly different between the *An. arabiensis *and *An. funestus *high and low transmission locations.

**Conclusion:**

These finding suggest that the abundance, distribution and malaria transmission of different malaria vectors are driven by different environmental factors. A better understanding of the specific ecological parameters of each malaria mosquito species will help define their current distributions, and how they may currently and prospectively be affected by climate change, interventions and other factors.

## Background

In sub-Saharan Africa, *Plasmodium falciparum *malaria is primarily transmitted by mosquito species belonging to the *Anopheles gambiae *and *Anopheles funestus *complexes [1-4]. The intensity of malaria transmission is heterogeneous across the continent, and influenced by mosquito species' compositions, vector competence, and underlying demographic and environmental factors [5]. High levels of transmission frequently occur where both *An. gambiae sensu lato *and *An. funestus *are present, as they tend to exploit different breeding habitats and peak at different times, thereby prolonging the transmission period. Generally, *Anopheles gambiae s.l*. are most abundant during the rainy season, and *An. funestus *is predominant at the end of the rains and beginning of the dry season [1-3]. The extent to which these species are influenced by the same environmental factors is largely unknown, as very few studies have examined them simultaneously over a wide geographical range. One of the most comprehensive studies undertaken in Kenya, by Mbogo *et al *[6], provides an opportunity to retrospectively analyse the spatial abundance, distribution and transmission data on *An. gambiae s.l*. and *An. funestus*, and compare climate, vegetation and elevation data derived from remote-sensed satellite sources in key locations.

The study by Mbogo *et al *[6] provides information on the numbers and transmission intensities, i.e. entomological inoculation rates (EIRs), of *An. gambiae s.l*. and *An. funestus *at 30 villages in the Malindi, Kilifi and Kwale Districts along the south-eastern coast of Kenya. Mosquito collections between June 1997 and May 1998 indicated that *An. gambiae sensu stricto, Anopheles arabiensis *and *An. funestus *were the main malaria vectors, with differing geographical abundance and transmission patterns over the 200 km study area. Interestingly, *An. gambiae s.s*. was found to be widespread, whereas *An. arabiensis *was mostly confined to Malindi in the north and *An. funestus *to Kwale in the south. Preliminary climate analyses by Mbogo *et al *[6], found positive correlations between rainfall and the temporal distributions of *An. gambiae s.l*. and *An. funestus*, however, these varied by species and between districts, and climate data were limited to one meteorological station in each district.

The recent advances in space technology and increased public access to remote-sensed satellite data provide a cost-effective and efficient alternative to examine relationships between climate, the environment and mosquito vectors of human disease [7,8]. This is important in poorly resourced regions of the world where the collection of reliable data over large geographical areas is not possible. As a follow-up to the Mbogo *et al *[6] study, comparisons of satellite-derived precipitation, temperature, humidity, vegetation and elevation measures at each study site in Malindi, Kilifi and Kwale Districts, and in *An. gambiae s.s., An. arabiensis *and *An. funestus *clustered locations were carried out.

## Methods

First, the average number and daily EIRs of *An. gambiae *s.l and *An. funestus *(data from Table 1 in Mbogo *et al *[6]), and climate, vegetation and elevation data for each district were summarized. Second, the relationship between the relative contribution (%) of the three main species, i.e. *An. gambiae *s.s, *An. arabiensis *and *An. funestus*, to annual EIR (data from Table 2 in Mbogo *et al *[6]), and each environmental variable was examined using bivariate correlations, and Pearson's correlation coefficient (2-tailed *P *value ≤ 0.05 significance). Third, the spatial patterns of *An. gambiae *s.s, *An. arabiensis *and *An. funestus *transmission were examined in ArcGIS using Spatial Analyst tools (ESRI, Redland, CA). The Moran's *I *statistic was used to determine spatial autocorrelation patterns i.e. clustered, dispersed, random, and the Getis-Ord Gi* statistic was to identify the locations with high and low clustering (Z scores, 95% confidence levels (CI) -1.96 and +1.96 standard deviations). The distributions of clustering across the study area were highlighted in relation to elevation, using a 3D wireframe map created in the surface mapping programme Surfer 7.0 (Golden Software Inc., Golden, CO). Mean environmental measures between high and low clustering trends were compared using the Mann-Whitney *U *test with Bonferroni correction for multiple comparisons. All statistical analyses were performed in Microsoft Excel and SPSS 16.0 (SPSS Inc., Chicago, IL).

**Table 1:** Bivariate correlations between *Anopheles *species and environmental variables

Environmental Variable	*An. gambiae *s.s	*An. arabiensis*	*An. funestus*
Precipitation	0.246	0.315	-0.550**
Temperature	-0.159	0.334	0.656**
Humidity	-0.159	0.334	0.656**
NDVI	0.217	0.031	0.392*
Elevation	-0.186	-0.370*	-0.046
*An. arabiensis*	0.024	-	-
*An. funestus*	-0.454*	-0.385*	

* Correlation is significant at ≤0.05 level (2-tailed)

** Correlation is significant at ≤0.01 level (2-tailed)

**Table 2:** Comparison of mean environmental measures between *An. gambiae *s.s, *An. arabiensis *and *An. funestus *high and low clustering trends

Environmental variable	*An. gambiae *s.s		*An. arabiensis*		*An. funestus*	
	High n = 17	Low n = 13	High n = 10	Low n = 20	High n = 11	Low n = 19
Precipitation	3.57	3.16*	3.77	3.2**	2.95	3.65**
Temperature	24.9	25.2*	24.7	25.2**	25.5	24.7**
Humidity	0.0166	0.0170	0.0164	0.0170	0.0174	0.0164**
NDVI	0.470	0.522	0.462	0.508	0.526	0.473
Elevation	54.6	78.1	11.4	91.5	104.4	41.8

Note. High = Z score>0, Low = Z score <0

* Significant difference at ≤0.05 level

**Significant difference at ≤0.0033 level after Bonferroni correction

Climate and vegetation data corresponding to the 30 mosquito collection sites (i.e. latitude and longitude), and original time period (i.e 1997-1998) were obtained from the best available sources, accessed via the IRI/LDEO Climate Data Library of the International Research Institute for Climate and Society [9]. Average daily precipitation (mm), monthly temperature (C°) and daily specific humidity (qa) measures for each month were extracted from satellite data from the National Oceanic and Atmospheric Administration (NOAA) [10-12]. Vegetation cover was based on Normalized Difference Vegetation Index (NDVI) satellite data extracted from monthly maximum NDVI data available from U.S Geological Survey's (USGS), Africa Data Dissemination Service [13]. Elevation data were derived from the USGS ETOPO2 Digital Elevation Model available from ESRI (Redlands, CA).

## Results

### District summaries

**Figure 1:**
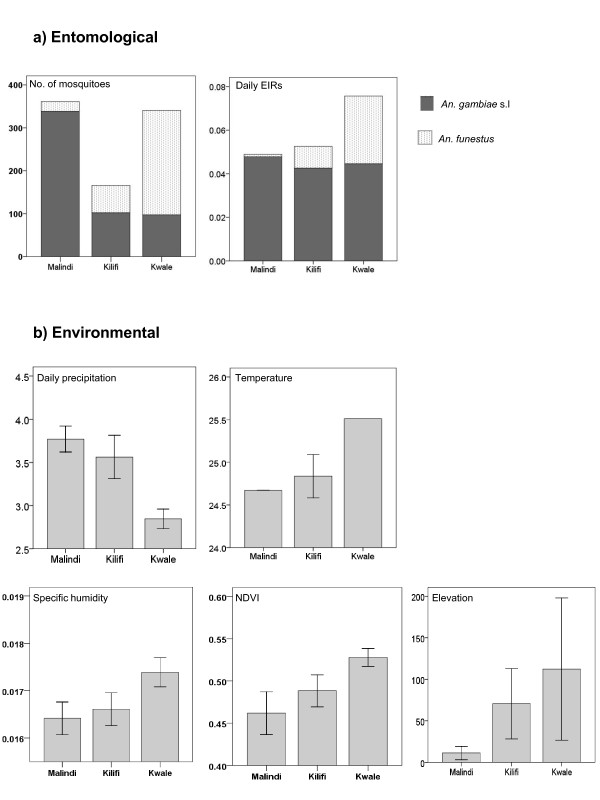
**Comparisons of mean entomological and environmental measures by district**. Note: Entomological data from Table 1 in Mbogo *et al *2003.

The findings of these analyses suggest that the different mosquito species compositions found in Malindi, Kilifi and Kwale Districts during 1997 and 1998 may be related to their different climate and topographical profiles. Figure 1 shows that the 10 sites from the Malindi District in the north, comprised predominately of *An. gambiae s.l*., had significantly (95% CI) higher precipitation, but lower temperature, specific humidity, NDVI and elevation measures than the 10 sites from Kwale District in the south, where *An. funestus *was most prevalent. Overall, these trends are supported by the correlations between the three main species, and each environmental variable (Table 1). *Anopheles gambiae *s.s. and *An. arabiensis *are positively correlated with precipitation, and negatively correlated with temperature and humidity measures. This contrasts to *An. funestus*, which was significantly negatively correlated with precipitation, but positively with temperature, humidity and NDVI. Interestingly, correlation analysis between each of these three *Anopheles *species, indicated that *An. gambiae *s.s (r = -0.454) and *An. arabiensis *(r = -0.385) were negatively correlated with *An. funestus*, which is in accordance with observations by Mbogo *et al *[6].

### Spatial analyses

**Figure 2:**
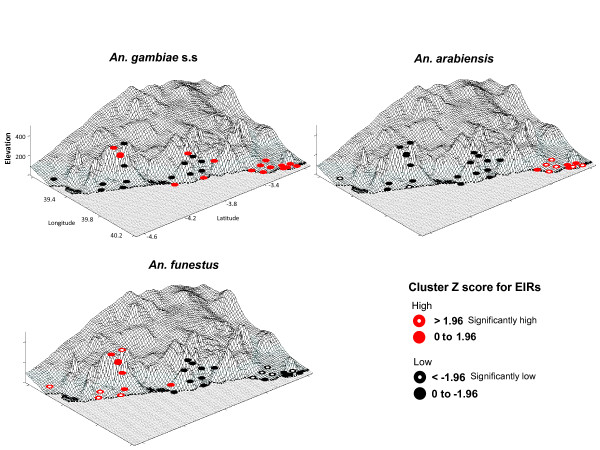
**Distribution of spatial clustering trends of high and low EIR values for *An. gambiae *s.s, *An. arabiensis *and *An. funestus***. Note: Z score > 0 indicates a clustering trend of high EIR values (red dots) and Z score < 0 indicates a clustering trend of low EIR values (black dots).

Spatial analyses indicated positive spatial autocorrelation or clustering for *An. arabiensis *(Moran's I value = 0.18, Z score = 3.8, *P *≤ 0.01) and *An. funestus *(MI = 0.24, Z score = 4.41, *P *≤ 0.01), but not for *An. gambiae s.s*. (MI = 0.03, Z score = 1, *P *≥ 0.05). The resultant Z scores of the Getis-Ord Gi* hot spot analyses (using inverse-distance weighting), indicated similar trends with significant spatial clusters of high and low EIR values found for *An. arabiensis *and *An. funestus *but not for *An. gambiae s.s*. The clustering trends are shown in Figure 2, and highlight the distinct patterns of each species across the study region. For *An. gambiae *s.s, 17 locations had positive Z scores (ranging 0.38 to 1.73) predominantly in the north, while the remaining 13 locations had negative Z scores (ranging -0.16 to -1.70) predominately in the south. For *An. arabiensis*, six locations with high EIR values were significantly clustered (Z scores ≥ 1.96) in Malindi District, and two with low EIR values (Z score ≤ -1.96) in Kwale District. This contrasts to *An. funestus*, which had five high EIR values significantly clustered in Kwale District, and five with low EIR values in Malindi District.

### Environmental comparisons

For each species, comparisons of environmental measures between locations with high and low transmission trends are shown in Table 2. Due to the small numbers in the study, and few locations with significant spatial clustering, these analyses were limited to mean comparisons between locations with high and low EIR clustering trends defined by positive Z scores (> 0) and negative Z scores (< 0), respectively. Overall, *An. gambiae *s.s and *An. arabiensis *showed similar environmental trends, with locations with higher transmission having higher precipitation, but lower temperature, humidity and NDVI measures than those locations with lower transmission by these species and/or where transmission by *An. funestus *was higher. Notably, locations with higher *An. arabiensis *transmission trends had markedly low elevations, also illustrated in Figure 2. Statistical comparisons indicated that for *An. gambiae *s.s there were no significant differences (*P *value <0.0033 Bonferroni corrected), while for *An. arabiensis *precipitation and temperatures were found to be significantly different, and for *An. funestus *precipitation, temperatures and humidity were found to be significantly different between the higher and lower transmission locations.

## Conclusion

These simple comparative analyses of 30 sites across three districts in Kenya indicate that *An. gambiae *s.l and *An. funestus *can have distinct ecological niches and requirements within a relatively small geographical area. This is supported by other entomological studies carried out in the region, which highlight the heterogeneous nature of these species' seasonality, host feeding preferences [14-16], body size [17] and the distribution and type of breeding sites [18-20]. For example, *An. gambiae *s.s larvae mostly occur in open shallow sunlit puddles and pools close to homesteads, whereas *An. funestus *larvae prevail in permanent vegetated aquatic habitats such as stream pools of rivers. In general, malaria transmission by *An. funestus *predominantly occurs in rural areas of sub-Saharan Africa, and the fact that Kwale District was less urbanized than the other districts [21,22], may also explain why *An. funestus *prevailed in this region. Furthermore, the presence of both *An. gambiae s.s*. and *An. funestus*, whose ecological requirements may be complementary to each other [23], may also account for the overall higher EIRs found in Kwale District [6].

Changes in the local environment are important to understand because they can create, or reduce the number of, suitable breeding sites for local vectors, thereby affecting their abundance and transmission patterns. In central Kenya, the introduction of irrigated rice cultivation appeared to reduce the risk of malaria transmission by *An. funestus *but not by *An. arabiensis *[24], and in Lake Victoria, a recent reduction in water level has created newly emerged land and habitats more suitable for *An. funestus *than for *An. gambiae *[25]. Along the Kenyan coast, information on the impact of urbanisation [22], agricultural activities and changes in climate on malaria transmission is limited, but becoming increasingly important. Currently, the prolonged drought affecting Kwale District and other Kenyan communities, has resulted in changes to human food security, population movement, cattle density, grazing and water storage practices [26,27], which will almost certainly alter vector abundance distributions and the risk of malaria.

Similarly, the impact of interventions such as insecticide-treated bed nets (ITNs), long-lasting insecticide-treated nets (LLINs) and indoor residual spraying (IRS) should be considered as they may affect species differently, especially if distributed widely over large geographical areas. The introduction of ITNs in Kilifi and Kwale District during the 1990s significantly reduced the number of indoor-resting *Anopheles *species, and a change in mosquito composition and biting times of *An. gambiae s.l*. [28], and in feeding preference of *An. funestus *with a shift among the outdoor resting females from endophagy on humans to exophagy on animals [15]. However, these ITNs were restricted to selected areas and are unlikely to have affected the overall relative abundance of the difference species in the study region. Other studies in East Africa [29,30] and elsewhere [31-33] have shown that *An. funestus *can be readily eliminated from an entire area by IRS programmes. However, this vector can reappear and become widespread again, sometimes with resistance to the insecticides used in the spray campaign [34-36]. This poses a further complication for vector control programmes. It also emphasizes the need for on-going mosquito and insecticide resistance surveillance [37], especially given the mass distribution of LLINs and IRS programmes currently taking place across sub-Saharan Africa, which could alter mosquito compositions and transmission dynamics over time [38].

Although there was considerable overlap between *An. gambiae s.s*. and *An. arabiensis*, *An. gambiae *s.s had no significant clustering or environmental differences between high and low transmission locations. The reasons for this may be related to its wide distribution and ability to exploit a range of habitats [1-3,18], but may also be because this species may comprise different molecular or chromosomal forms which are not well defined in this region compared with other regions of sub-Saharan Africa [1]. In West Africa, the chromosomal forms of *An. gambiae *s.s have shown to have differing spatial distributions and environmental parameters [39-41], and distinct differences between the M and S molecular forms have been described in Mali [42]. In this coastal region of Kenya, only the *An. gambiae *S form has been detected in two locations [43], therefore, a better understanding of the speciation and transmission patterns of the *An. gambiae *s.s forms is crucial, especially as *An. gambiae *s.l appears to be the main vector of both malaria and lymphatic filariasis in Kilifi and Kwale Districts [15,44,45].

The study by Mbogo *et al *[6] collected mosquitoes using pyrethroid spray catches (PSC) inside houses, which could potentially underestimate the abundance of exophilic mosquito species such as *An. arabiensis*, as shown in other East African countries [46]. In general, measuring the population dynamics of *An. gambiae *s.l and *An. funestus *is difficult, and studies have shown great variability depending on the sampling technique used, and whether interventions such as ITNs are present and acting as a deterrent [16,28,47-49]. The presence of cattle for *An. arabiensis *is also an important consideration as they prefer to feed on these animals over humans and other livestock [46,50,51]. Although there are limitations to using the PSC method to estimate abundance and transmission patterns, the study by Mbogo *et al *[6] is one of largest datasets available for East Africa, which compares the abundance and transmission potential of *An. gambiae *s.l and *An. funestus *across a diverse ecological range using a standard sampling technique.

Malaria transmission is complex, and more knowledge on the relationship between the environment, mosquito vectors, human disease and demography in sub-Saharan Africa will help implement appropriate control measures in a rapidly changing landscape. This is particularly important in areas already reporting changes in transmission intensity [52,53], and may be additional factors to include in future malaria models. This small follow-up study to Mbogo *et al *[6] aimed to elucidate environmental factors associated with the *An. gambiae *and *An. funestus *complexes in one region of Kenya. It exemplifies what can be done with existing entomological data contained in the literature and elsewhere, and how modern satellite and GIS technologies in public health research may be exploited, especially for climate sensitive diseases in developing countries, such as malaria [54,55].

## Competing interests

The authors declare that they have no competing interests.

## Authors' contributions

LKH identified data sources, designed the study, carried out the data analysis and wrote the first draft of the manuscript. JH participated in the interpretation of the results and editing of the manuscript. EM conceived the idea for the study, and contributed to the writing and editing of the manuscript. All authors have read and approved the final manuscript.
